# Facile Multiple Alkylations of C_60_ Fullerene

**DOI:** 10.3390/molecules27020450

**Published:** 2022-01-10

**Authors:** Kazuhira Miwa, Shinobu Aoyagi, Takahiro Sasamori, Shogo Morisako, Hiroshi Ueno, Yutaka Matsuo, Hideki Yorimitsu

**Affiliations:** 1 Department of Information and Basic Science, Graduate School of Science, Nagoya City University, Nagoya 467-8501, Aichi, Japan; gs177706@nsc.nagoya-cu.ac.jp (K.M.); morisako.shogo.gf@u.tsukuba.ac.jp (S.M.); 2 Tsukuba Research Center for Energy Materials Sciences (TREMS), Division of Chemistry, Faculty of Pure and Applied Sciences, University of Tsukuba, 1-1-1 Tennodai, Tsukuba 305-8571, Ibaraki, Japan; 3 Creative Interdisciplinary Research Division, Frontier Research Institute for Interdisciplinary Sciences and Department of Chemistry, Graduate School of Science, Tohoku University, Sendai 980-8578, Miyagi, Japan; hiroshi.ueno.d5@tohoku.ac.jp; 4 Department of Chemical System Engineering, Graduate School of Engineering, Nagoya University, Nagoya 464-8603, Aichi, Japan; yutaka.matsuo@chem.material.nagoya-u.ac.jp; 5 Department of Mechanical Engineering, School of Engineering, The University of Tokyo, Tokyo 113-8656, Japan; 6 Department of Chemistry, Graduate School of Science, Kyoto University, Kyoto 606-8502, Japan; yori@kuchem.kyoto-u.ac.jp

**Keywords:** fullerene, C_60_, alkylation, sodium dispersion, dialkyl sulfate

## Abstract

The reduction of fullerene (C_60_) with sodium dispersion in the presence of an excess amount of dipropyl sulfate was found to yield highly propylated fullerene, C_60_(*n*C_3_H_7_)_n_ (max. *n* = 24), and C_60_(*n*C_3_H_7_)_20_ was predominantly generated as determined by mass spectroscopy.

## 1. Introduction

**Figure 1:**
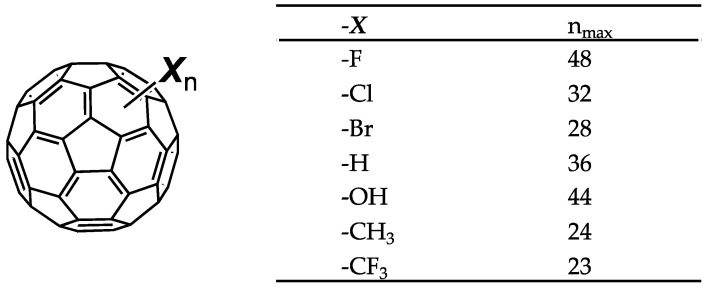
Reported multiply functionalized fullerenes C_60_*X*_n_ (*n* > 20) [10,11,12,13,14,15,16].

The discovery of fullerenes has created opportunities regarding new chemistries of unique three-dimensional π-electron conjugated systems [1]. In particular, the functionalization of fullerenes has considerably promoted fullerene chemistry in various fields [2,3,4,5,6,7,8]. In this regard, the introduction of functional groups to the fullerene surface as a prompt functionalization of fullerenes should be of great interest. Because of the strained geometry of aromatic systems, the π-bonds of a fullerene should be more reactive than those of planar arene-type aromatic compounds. For example, mono-functionalized fullerenes, RC_60_H (R: aryl and alkyl groups), have been obtained by the mechanochemical reaction of C_60_ with aryl and alkyl bromides in the presence of alkali metals under solvent-free reaction conditions [9]. Moreover, multiply functionalized fullerenes, C_60_*X*_n_, have been reported (Figure 1) [10,11,12,13,14,15,16]. The addition reactions of the C=C π-bonds of a fullerene have been reported to generate polyhalogenated fullerenes [17,18,19,20,21,22]. Using multiply halogenated fullerenes, nucleophilic substitutions of polyhalogenated fullerenes with anionic organometallic species, such as methyllithium or phenyllithium, were investigated to obtain the corresponding multiply alkylated/arylated fullerenes [23,24,25]. Multiply arylated fullerenes, C_60_(H-Ar)_n_, can also be obtained by the AlCl_3_-catalyzed Friedel–Crafts reaction of C_60_ with aromatics, which is characterized as the fullerenation of aromatics [26,27]. Alternatively, the reactions of metastable poly-anions of fullerene with electrophiles could be an accessible methodology for the introduction of substituents on a fullerene framework, because fullerenes are known to be good electron acceptors due to their low reduction potentials [15,28,29]. For example, when the corresponding polyanionic fullerene generated by reduction with lithium was trapped by an excess amount of CH_3_I to produce highly methylated fullerenes, the most methylated product was C_60_(CH_3_)_24_ [15]. However, the predominant components were C_60_(CH_3_)_6_ and C_60_(CH_3_)_8_, and a variety of methylated fullerenes, i.e., a series of C_60_(CH_3_)_n_ (0 ≤ *n* ≤ 24), were formed due to the lability of the polyanionic fullerene. We present an efficient multiple-alkylation reaction of fullerene (C_60_) using an *in situ* reduction/substitution reaction system using sodium dispersion (SD) and dipropyl sulfate (DS). SD is a well-known, effective reductant [30,31,32,33,34,35,36], and DS is an electrophile that is inert to SD. Thus, the coexistence of SD and DS allows the reactant to undergo repetitive tandem reduction/substitution reactions *in situ*.

## 2. Results and Discussion

### 2.1. Synthesis and Separation of C_60_(nC_3_H_7_)_n_

**Scheme 1:**
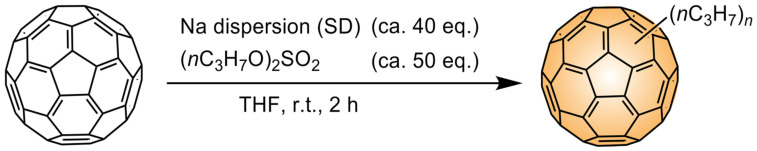
Reaction of C_60_ with SD in the presence of DS.

**Figure 2:**
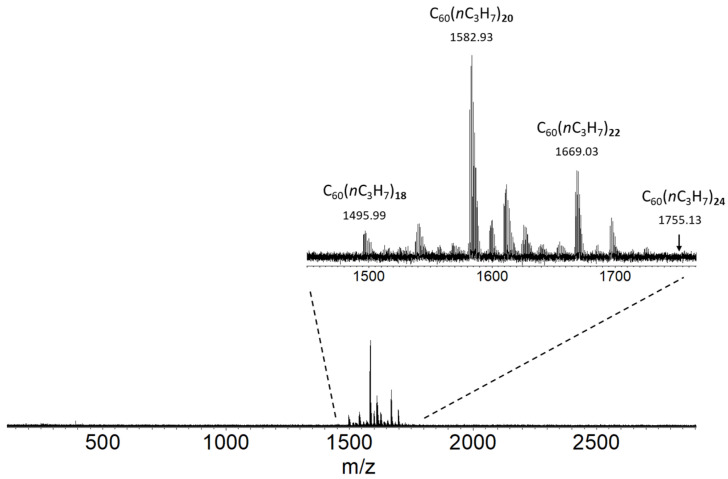
DART-MS (positive) of the obtained mixture of C_60_(*n*C_3_H_7_)_n_.

The reduction of fullerene, C_60_ (25.2 mg, 35.0 μmol), with an excess amount of SD (150 μL, ca. 40 eq.) in the presence of an excess amount of DS (290 μL, 1.75 mmol, ca. 50 eq.) in THF (2 mL) at room temperature (r.t.) resulted in the formation of a mixture of multiply *n*-propylated fullerenes, C_60_(*n*C_3_H_7_)_n_ (Scheme 1). Although the number (n) of propyl groups introduced to the fullerene varied to some degree, as shown by the direct analysis in real time mass spectrum (DART-MS) in Figure 2, the distribution of n was remarkably narrower and the substitution numbers (n) of the predominant components were larger, C_60_(*n*C_3_H_7_)_20_ (*n* = 20), relative to the previous case of methylation [15]. The obtained mixture mainly consisted of components with *n* = even number (18, 20, and 22). A small peak observed around *n* = 19 (*m*/*z* = 1539) can be explained by the E2-type reaction of the anionic intermediate (C_60_(*n*C_3_H_7_)_19_^−^) with DS or the hydrogen abstraction of the radical intermediate (C_60_(*n*C_3_H_7_)_19_) from DS and/or the solvent (THF) to form C_60_(*n*C_3_H_7_)_19_H (*m*/*z* = 1540), instead of the expected S_N_2-type reaction with DS to form C_60_(*n*C_3_H_7_)_20_. Some small peaks observed between *n* = 20 and 22 (*m*/*z* = 1583 and 1669) can most likely be interpreted as the occasional oxidation of C_60_(*n*C_3_H_7_)_20_ in the air to form C_60_(*n*C_3_H_7_)_20_O_m_ (*m*/*z* = 1583 + 16 m).

**Figure 3:**
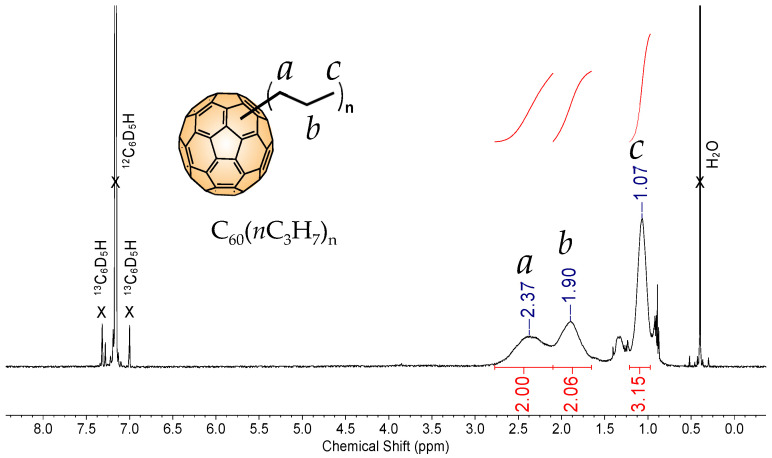
^1^H NMR (500 MHz, C_6_D_6_) spectrum of the obtained mixture of C_60_(*n*C_3_H_7_)_n_.

**Figure 4:**
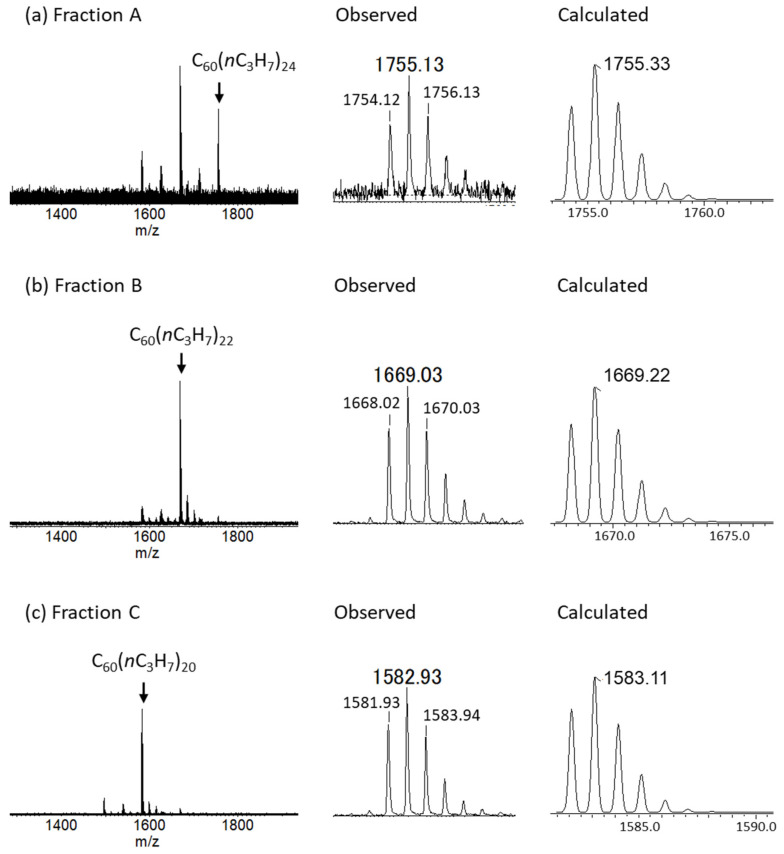
DART-MS (positive) of Fractions **A**, **B**, and **C** obtained through column chromatography (SiO_2_, eluent: *n*-hexane), the predominant components of which are (**a**) C_60_(*n*C_3_H_7_)_24_, (**b**) C_60_(*n*C_3_H_7_)_22_, and (**c**) C_60_(*n*C_3_H_7_)_20_, respectively. **Left**: observed whole spectra of each C_60_(*n*C_3_H_7_)_n_ fraction. Middle: enlarged isotopic patterns of the corresponding C_60_(*n*C_3_H_7_)_n_ (*n* = 24, 22, 20). **Right**: simulated isotopic patterns of C_60_(*n*C_3_H_7_)_n_ (*n* = 24, 22, 20) as [M + H]^+^.

The ^1^H NMR (500 MHz, C_6_D_6_) spectrum of the mixture of multiply *n*-propylated fullerene (Figure 3) revealed three broad peaks at 2.37 (*a*), 1.90 (*b*), and 1.07 (*c*) ppm, attributed to the propyl chain of C_60_(*n*C_3_H_7_)_n_. The peaks broadened with down-fielded chemical shifts from *c* for the terminal methyl group to *a* for the methylene group attached to C_60_. The peak broadening would be caused by the existence of various attached propyl groups with slightly different chemical shifts as the mixture contained several C_60_(*n*C_3_H_7_)_n_ with different substitution numbers (n) and their structural isomers. Notably, a certain amount of the multiply propylated fullerenes could be obtained by the one-pot reaction, albeit as an inseparable mixture. Thus, the present reaction could be a viable method to obtain multiply alkylated fullerenes in a one-pot reaction. After the partial purification of the crude mixture (180 mg) through column chromatography (SiO_2_, eluent: *n*-hexane, length/diameter: 25 cm/20 mmφ), three fractions of yellow powders were separately obtained, in which the predominant component of each fraction was C_60_(*n*C_3_H_7_)_24_ (Fraction **A**, 12 mg), C_60_(*n*C_3_H_7_)_22_ (Fraction **B**, 3 mg), and C_60_(*n*C_3_H_7_)_20_ (Fraction **C**, 21 mg) (Figure 4). *n*-Hexane was found to be a better eluent than the other polar solvents in purification. Among the obtained fractions, the most propylated product was C_60_(*n*C_3_H_7_)_24_, which had the same number as that of the most methylated product obtained by reduction with Li followed by the addition of CH_3_I in the previous report [15].

### 2.2. Characterization of C_60_(nC_3_H_7_)_n_

**Figure 5:**
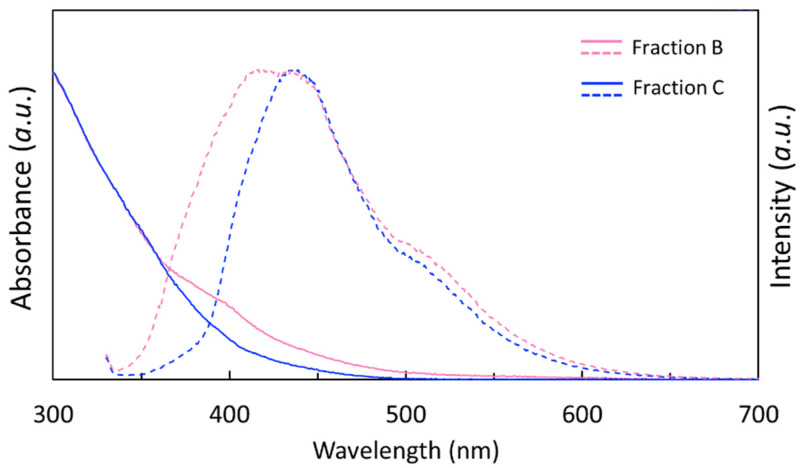
UV/vis absorption and fluorescence spectra of Fraction **B** [C_60_(*n*C_3_H_7_)_22_] (pink) and Fraction **C** [C_60_(*n*C_3_H_7_)_20_] (blue). The solid and dashed lines are the UV/vis absorption and fluorescence spectra of each fraction in *n*-hexane, respectively.

**Figure 6:**
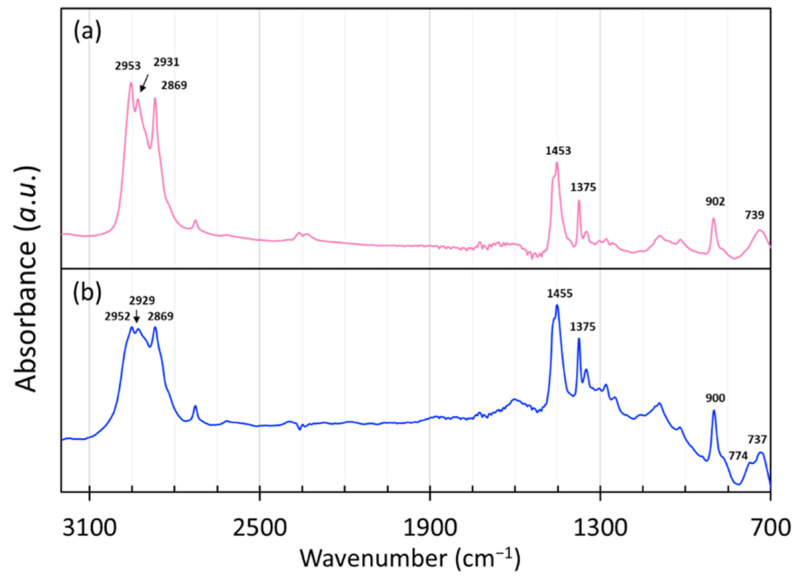
IR spectra of (**a**) Fraction **B** [C_60_(*n*C_3_H_7_)_22_] and (**b**) Fraction **C** [C_60_(*n*C_3_H_7_)_20_].

The UV/vis absorption and fluorescence spectra of the obtained Fractions **B** and **C** are shown in Figure 5. Fraction **A** [C_60_(*n*C_3_H_7_)_24_] was not used in this measurement because it clearly contained C_60_(*n*C_3_H_7_)_22_, as shown in Figure 4. Both fractions were found to fluoresce, at λ_max_ = 412 and 435 nm for Fraction **B** [C_60_(*n*C_3_H_7_)_22_], and 436 nm for Fraction **C** [C_60_(*n*C_3_H_7_)_20_] (excited: 300 nm). The fluorescence spectrum of Fraction **B** [C_60_(*n*C_3_H_7_)_22_] is considerably broader in the short-wavelength region compared with that of Fraction **C**, most likely due to the more contracted π-electron conjugation of C_60_(*n*C_3_H_7_)_22_ compared with that of C_60_(*n*C_3_H_7_)_20_. Considering both the absorption/fluorescence spectra of both fractions, the π-electron systems of fullerene would be partially contracted but preserved on the spherical skeleton of C_60_, to some degree. The IR spectra of Fraction **B** [C_60_(*n*C_3_H_7_)_22_] and Fraction **C** [C_60_(*n*C_3_H_7_)_20_] are shown in Figure 6. The two spectra are similar, and the most intense band, 2800–3000 cm^−1^, is assignable to the C–H stretch modes of the propyl chains. The moderate-intensity bands at around 1450 and 1375 cm^−1^ can be assigned to the C–H scissoring and C–H methyl rock modes, respectively.

**Figure 7:**
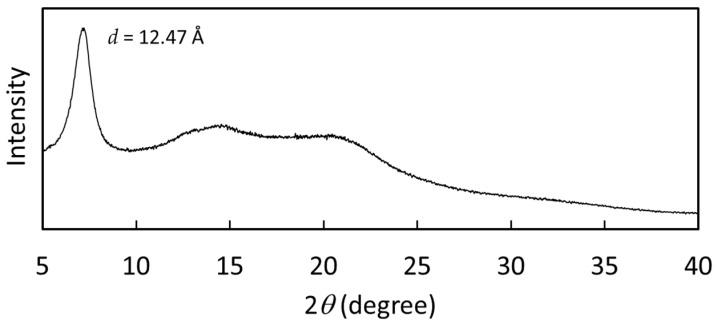
Powder X-ray diffraction of Fraction **C** [C_60_(*n*C_3_H_7_)_20_].

Figure 7 illustrates the X-ray powder diffraction pattern of Fraction **C** [C_60_(*n*C_3_H_7_)_20_] at room temperature (X-ray wavelength: CuKα 1.54 Å). Three broad amorphous peaks were observed in the pattern. The *d*-spacing value of the strongest peak at 2*θ* = 7.08° was 12.47 Å, which was larger than the intermolecular distance in solid C_60_ (ca. 10.0 Å) [37] and suggests that the intermolecular distance of the product was increased by the 20 attached propyl groups. The amorphous powder X-ray diffraction pattern suggests that the presence of structural isomers of C_60_(*n*C_3_H_7_)_20_ prohibited its crystallization. Isolation of the structural isomers using high-performance liquid chromatography (HPLC) and their crystallization using solvent vapor annealing [38] and other methods are required to determine their molecular structures in the next step.

### 2.3. Reaction Mechanism

**Scheme 2:**
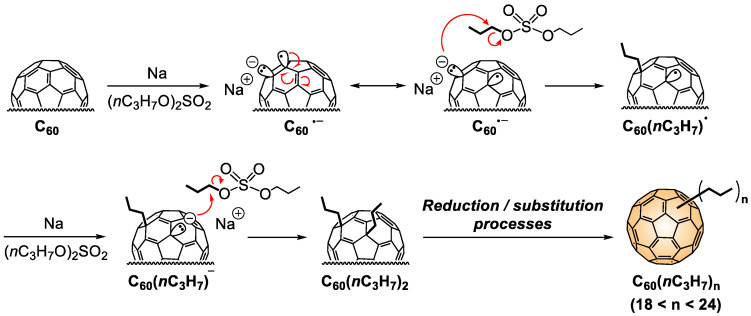
Plausible mechanism of the tandem propylation processes of C_60_.

The efficient formation of multiply propylated fullerenes can most likely be interpreted in terms of repetitive tandem reduction/substitution processes (Scheme 2). Considering the traditional reduction mechanism of aromatic compounds, in the initial step, C_60_ could be reduced through SD to provide the fullerene radical anion C_60_^−^ by one-electron reduction. Because of the π-electron conjugated framework of fullerene, the generated radical anion species should be stabilized. Then, the generated C_60_^−^ would undergo nucleophilic substitution toward DS, yielding the radical C_60_(*n*C_3_H_7_), which would be easily reduced in the next step to yield the corresponding anion, C_60_(*n*C_3_H_7_)^−^. The unpaired electron in the radical C_60_(*n*C_3_H_7_) would be delocalized over five carbon atoms in the two six-membered rings adjacent to the carbon atom attached to the propyl group [39,40]. The subsequent nucleophilic substitution reaction would yield C_60_(*n*C_3_H_7_)_2_. Thus, the introduction of the two alkyl groups to the fullerene framework could occur via such a tandem reduction/substitution processes. Subsequently repeating the reduction/substitution processes could result in the formation of C_60_(*n*C_3_H_7_)_n_ (*n* = even number). Because the polyanionic species of fullerene are generally expected to be highly reactive/unstable, the repetition of the tandem reduction/substitution processes can be effective due to the easy trapping of anionic species generated in situ by the reduction-resistant electrophile, DS. The use of DS instead of conventional propylating agents, such as propyl iodide, is the key to success because the former does not undergo single-electron reduction with SD, while the latter does very easily. However, with increases in n, steric hindrance could hamper the nucleophilic substitution processes. Considering the plausible formation mechanism of C_60_(*n*C_3_H_7_)_n_, the formation of C_60_(*n*C_3_H_7_)_19_H with an odd number of propyl groups can likely be explained by the E2-type reaction of the in situ generated radicals and/or anionic species of C_60_(*n*C_3_H_7_)_19_ with DS, rather than the slow nucleophilic substitution process.

### 2.4. Application to Multiple Methylation

**Figure 8:**
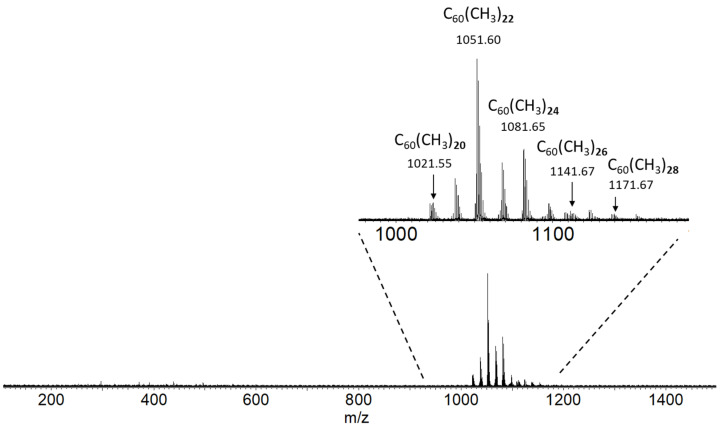
DART-MS (positive) spectrum of the obtained mixture of C_60_(CH_3_)_n_.

The abovementioned method was also applied to the synthesis of methylated fullerenes using dimethyl sulfate. The reaction of C_60_ with SD in the presence of an excess amount of a less hindered electrophile, dimethyl sulfate, was conducted under the same conditions, expecting to form a more highly methylated fullerene, C_60_(CH_3_)_n_. Consequently, the number of methyl groups ranged from *n* = 20 to 28 (Figure 8). Peaks with *n* = odd number, which would be caused by C_60_(CH_3_)_n_H and C_60_(CH_3_)_n−1_O, were observed more clearly in methylation (Figure 8) than in propylation (Figure 2). The E2-type reaction and the oxidation reaction to form the hydrogenated and oxidized products, respectively, would be inhibited in propylation by severe steric hindrance due to the attached propyl groups relative to methylation. The larger steric hindrance of propyl groups could also decrease the number of possible structural isomers of the products. However, the maximum number of n was higher in methylation with smaller methyl groups. Notably, even with an efficient alkylation method, the most methylated product observed by DART-MS was C_60_(CH_3_)_28_, implying that further reduction of the 28-methylated fullerene could be difficult owing to the large number of electropositive alkyl groups attached to the fullerene framework and the associated contraction of its π-electron conjugated systems. Using SD and dialkyl sulfate, the number of alkyl groups introduced to fullerene increased, and the distribution of their number (n) became considerably narrower relative to the previously reported cases.

## 3. Materials and Methods

### 3.1. General Information

All manipulations were performed under an argon atmosphere using Schlenk line techniques. SD and DS were purchased from Tokyo Chemical Industry Co., Ltd. (TCI, Tokyo, Japan). Anhydrous THF and dimethyl sulfate were purchased from FUJIFILM Wako Pure Chemical Corporation.

Mass spectra were obtained using a JEOL JMS-T100LP (DART) mass spectrometer. The ^1^H NMR (500 MHz) spectrum was measured using a JEOL ECZ-500R spectrometer. UV/vis absorption and fluorescence spectra were recorded using a JASCO V-770 spectrophotometer and FP-8500 spectrofluorometer, respectively. IR spectra were recorded using a Bruker LUMOS FTIR microscope. Powder X-ray diffraction data were recorded using a Rigaku MiniFlex600 diffractometer.

### 3.2. Reactions of C_60_ with SD in the Presence of DS

A Schlenk tube was charged with SD (150 μL, ca. 40 eq.). The SD was washed with *n*-hexane (2 mL × 3) to remove the mineral oil and dried under reduced pressure to remove the remaining solvent. Then, THF (2 mL), DS (290 μL, 1.75 mmol, ca. 50 eq.), and C_60_ (25.2 mg, 35.0 μmol) were added, and the resulting suspension was vigorously stirred at room temperature for 2 h. The color of the reaction mixture changed from brown to yellow. Sodium ethoxide, which was prepared from sodium and ethanol, was then added slowly and the resulting suspension was stirred at 50 °C for an additional 2 h. H_2_O was used to stop the reaction, and the resulting biphasic solution was extracted using toluene. The organic phase was washed with *sat.* NH_4_Claq and *sat.* NaHCO_3_aq, dried over MgSO_4_, filtered, and concentrated under reduced pressure. The residue was purified on a silica gel column using *n*-hexane to provide C_60_(*n*C_3_H_7_)_n_ as a yellow solid.

### 3.3. Reactions of C_60_ with SD in the Presence of Dimethyl Sulfate

A Schlenk tube was charged with SD (150 μL, ca. 40 eq.). The SD was washed with *n*-hexane (2 mL × 3) to remove the mineral oil and dried under reduced pressure to remove the remaining solvent. Then, THF (2 mL), dimethyl sulfate (160 μL, 1.75 mmol, ca. 50 eq.), and C_60_ (25.5 mg, 35.0 μmol) were added, and the resulting suspension was vigorously stirred at room temperature for 2 h. The color of the reaction mixture changed from brown to yellow. After gradually adding 2-propanol to the reaction mixture, 2 M NaOHaq (2 mL) was added subsequentially, and the resulting biphasic solution was extracted using toluene. The organic phase was washed with brine, filtered, and concentrated under reduced pressure to provide C_60_(CH_3_)_n_ as a yellow solid.

## 4. Conclusions

We found that the reaction of fullerene with an excess amount of SD in the presence of DS yielded highly propylated fullerenes, C_60_(*n*C_3_H_7_)_n_ (18 ≤ *n* ≤ 24), and C_60_(*n*C_3_H_7_)_20_ and C_60_(*n*C_3_H_7_)_22_ were predominantly obtained with a small dispersion of *n* values. The presence of the reducing agent (SD) and electrophile (DS), which was inert to the reducing agent, allowed for repetitive reduction/substitution processes in which the reactive anionic intermediate could be easily trapped by the electrophile as soon as it was generated. It can be concluded that the multiple tandem alkylation reaction of fullerene is an effective method for producing highly alkylated fullerenes.

## Data Availability

Data available in a publicly accessible repository.
